# Haemophilus influenzae Epiglottitis: A Rare Disease Not to Be Forgotten

**DOI:** 10.7759/cureus.101680

**Published:** 2026-01-16

**Authors:** Madalena Ferreira, Luzia Condessa, Margarida Roquette, Rita Antão, Carina Cardoso, Margarida Chaves

**Affiliations:** 1 Pediatrics, Hospital de Cascais Dr. José de Almeida, Lisbon, PRT

**Keywords:** epiglottitis, haemophilus infuenzae, hib vaccine, non-typable haemophilus influenzae, upper airway obstruction

## Abstract

Although pediatric epiglottitis has become rare following the widespread use of the *Haemophilus influenzae* type b (Hib) vaccine, breakthrough infections still occur, whilst other *H. influenzae* serotypes continue to emerge. We present the case of a 10-year-old child, fully immunized against Hib, who developed epiglottitis. Blood cultures confirmed *H. influenzae *infection, and further microbiological characterization identified a non-typable strain. This case highlights the growing clinical relevance of *H. influenzae* strains not covered by the Hib vaccine and reinforces the need to maintain a high index of suspicion for epiglottitis as a cause of upper airway obstruction, even in fully immunized children, requiring early recognition and prompt management. Continuous monitoring is crucial for assessing how vaccination influences the epidemiological patterns of invasive *H. influenza*e disease.

## Introduction

Epiglottitis is a medical emergency, requiring early diagnosis and timely management [1-3]. It causes a life-threatening upper airway obstruction due to severe swelling of the epiglottis and nearby structures, which can lead to respiratory failure, sudden cardiopulmonary arrest, and death [3]. Epiglottitis etiologies are primarily infectious, after direct invasion by commensal pathogenic agents from the posterior nasopharynx and/or bacteremia [4]. Prior to vaccination, *Haemophilus influenzae* type b (Hib) was the most frequent cause of infection, mainly affecting children under five years of age [5-7].

However, with the widespread introduction of the Hib conjugated vaccine in the early 1990s, there was a dramatic decline in invasive Hib disease, including epiglottitis [5-12]. The 2023 Active Bacterial Core Surveillance Report by the CDC estimated annual rates of Hib infection of 0.22 cases per 100,000 among preschool children, contrasting with previous rates prior to vaccination, which could reach more than 20 cases per 100,000 [9-11]. This led to a shift towards *H. influenzae* strains not covered by the vaccine, such as non-type b serotypes and non-typable variants, along with an increasing prevalence among older children, adolescents, and adults rather than preschool-aged children, even in fully immunized patients [7,12-14].

Based on its capsular polysaccharide antigens, *H. influenzae* can be classified into six typable strains (a, b, c, d, e, f) and non-typable strains [13]. *Haemophilus influenzae* type b remains the only strain covered by current vaccination programs. Besides* H. influenzae*, other bacteria (*Streptococcus pneumoniae*, *Staphylococcus aureus*, and *Streptococcus pyogenes*) and viral agents (influenza A and B, parainfluenza, Epstein-Barr virus, or COVID-19) have also been associated with epiglottitis [14-17].

## Case presentation

**Figure 1:**
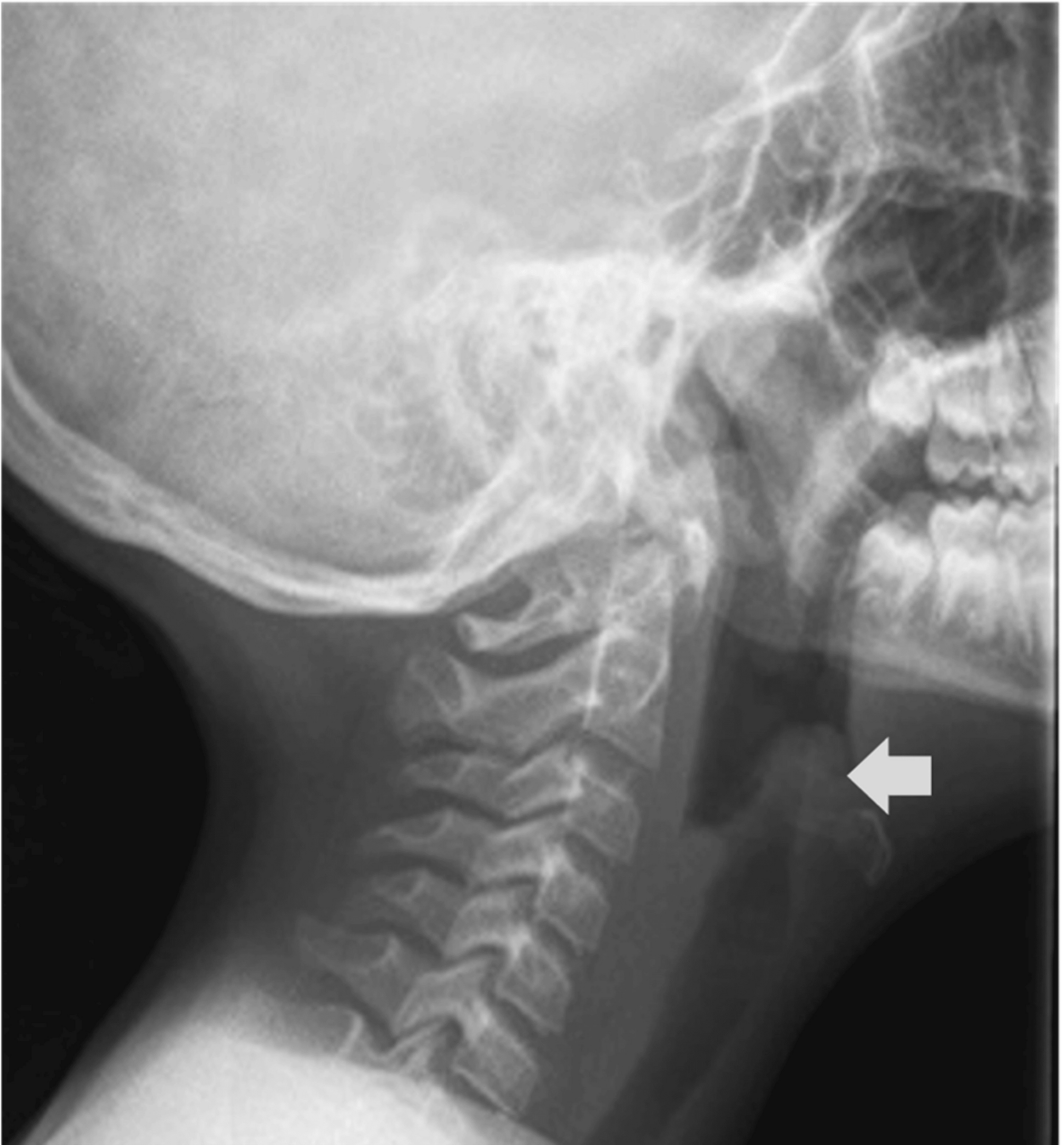
Lateral neck radiograph demonstrating the thumbprint sign The enlarged, edematous epiglottis (white arrow) produces a characteristic thumbprint appearance, consistent with acute epiglottitis.

A previously healthy 10-year-old boy presented to the emergency department with an acute onset of high fever, odynophagia, chest pain, and dyspnea. His clinical history revealed a complete vaccination schedule, including four doses of the Hib vaccine, and no known epidemiological context. This patient was in respiratory distress, febrile, and had a toxic appearance. He would avoid the supine position while sticking his tongue out and drooling, although oxygen saturation was >96% and lung sounds were clear to auscultation bilaterally. Given a heart rate of 130 bpm and a capillary refill time of three seconds, volume resuscitation was initiated, together with high-flow oxygen therapy, nebulized adrenaline, and ceftriaxone. Physical examination also revealed red and enlarged tonsils. Initial laboratory assessment (Table 1) showed leukocytosis (21,200/μL), neutrophilia (18,630/μL), elevated C-reactive protein (32.76 mg/dL), and procalcitonin (0.76 ng/mL). The chest radiograph was unremarkable; however, the lateral neck radiograph showed an enlarged epiglottis, creating the classic 'thumbprint' sign (Figure 1).

**Table 1:** Initial laboratory findings demonstrating markers of infection N/A: Not applicable

Laboratory parameters	Day one	Reference values
Haemoglobin (g/dL)	13.6	11.5-15.5
Leukocytes (/μL)	21,200	5,000-13,000
Neutrophils (/μL)	18,630	2,000-8,000
Platelets (/μL)	305,000	180,000-400,000
C-reactive protein (mg/dL)	32.76	<0.50
Procalcitonin (ng/mL)	0.76	<0.10
Microbiology		
Blood culture	*H. infuenzae:* Sensitivity to ampicillin and cefotaxime; sensitivity with increased exposure to amoxicillin-clavulanate	N/A

After stabilization, the otorhinolaryngologist was able to perform indirect laryngoscopy, revealing inflammation and edema of supraglottic structures without glottic obstruction. Later admitted into an intermediate care unit under continuous cardiorespiratory monitoring, the patient received IV ceftriaxone, clindamycin, prednisolone, and nebulized adrenaline. Isolation of non-typable *H. influenzae* in blood culture confirmed the diagnosis. Following progressive clinical and laboratory improvement, the patient was discharged after seven days and completed three additional days of oral amoxicillin-clavulanate with a good response to treatment.

## Discussion

Although epiglottitis among children has become a rare and often underrecognized diagnosis, this case of non-typable *H. influenzae* epiglottitis in a fully vaccinated 10‑year‑old boy underscores the evolving epidemiology and shifting pathophysiology in the post‑Hib vaccination era. It also highlights the potential reemergence of this life‑threatening disease with the need for a high index of suspicion to ensure early diagnosis and management.

Epiglottitis is diagnosed primarily through clinical assessment, emphasizing the importance of early symptom recognition. A Hib epiglottitis typically presents acutely, with a sudden onset and rapidly worsening symptoms [1-4]. Common manifestations include a toxic appearance, respiratory distress, high fever, sore throat, dysphagia, muffled voice, drooling, and stridor. In order to maintain a patent airway, affected children often assume the characteristic tripod position, i.e., sitting upright, leaning forward with a hyperextended neck [1-4]. In this case, the patient’s main complaints upon admission were acute chest pain and shortness of breath. He did not exhibit a muffled voice or stridor. However, suspicion of epiglottitis arose due to the combination of high fever, septic appearance, respiratory distress, sore throat, and tripod positioning.

Imaging is not essential for diagnosis and should not delay treatment, but it may help when there’s uncertainty [4]. In this case, the lateral neck radiograph showed a thickened epiglottis drawing the thumbprint sign: a thickened, round, radiopaque shadow resembling a thumb (Figure 1) [2]. 

Prompt stabilization and airway management are essential in patients with epiglottitis, given the risk of imminent upper airway obstruction [1-6]. In this case, the child received immediate support using a high‑flow oxygen mask, and because the airway remained stable with no signs of deterioration, emergent intubation was not required. Continuous monitoring was ensured, and an experienced airway management team was placed on standby. Volume resuscitation and broad‑spectrum intravenous antibiotics were initiated without delay. As recommended and considering the spectrum of potential pathogens (*H. influenzae*, *S. pneumoniae*, *S. pyogenes*, and *S. aureus*), the patient was started on a third‑generation cephalosporin (ceftriaxone) combined with an antistaphylococcal agent providing methicillin-resistant *S. aureus* (MRSA) coverage (clindamycin) [13].

The definitive diagnosis of epiglottitis requires microbiological confirmation, typically obtained through blood cultures or, when feasible, epiglottic culture [4]. However, this procedure carries a substantial risk of airway compromise and is generally reserved for patients who are already intubated. In this case, blood cultures identified a non-typable *H. influenzae* strain, which is not covered by Hib vaccination, underscoring the role of non-vaccine strains as potential causative agents of epiglottitis in fully immunized children.

This case occurred in Portugal, where Hib vaccination coverage exceeds 95%, and the vaccine has contributed to herd immunity, extending protection even to unvaccinated individuals [18]. Nonetheless, a 12‑year multicenter pediatric survey conducted in Portugal revealed a statistically significant increase in the incidence rate of invasive *H. influenzae* disease during the last six years (2016-2021), affecting all pediatric age groups but occurring most frequently in children under five years of age [18]. Overall, this case highlights the importance of maintaining clinical vigilance and ongoing microbiological surveillance to better characterize the evolving epidemiology of invasive* H. influenzae* disease.

## Conclusions

Epiglottitis remains a critical, life‑threatening condition that demands early recognition, timely airway management, and prompt antibiotic therapy. Since the introduction of the Hib conjugate vaccine, pediatric epiglottitis has become rare, often posing a diagnostic challenge in the modern era. This case highlights that *H. influenzae* breakthrough infections may still occur in fully immunized children due to the emergence of non‑type b strains. Therefore, clinicians must remain highly vigilant, particularly in children who exhibit symptoms of impending upper airway obstruction. Furthermore, this case highlights the need for ongoing epidemiologic surveillance to track the prevalence of emerging *H. influenzae* serotypes in the post-vaccination era. Continued investigation of serotype replacement and vaccine coverage is vital to clarify the changing pattern of invasive *H. influenzae* disease and to guide future vaccine policy decisions.
